# Predicting stress, strain and deformation fields in materials and structures with graph neural networks

**DOI:** 10.1038/s41598-022-26424-3

**Published:** 2022-12-17

**Authors:** Marco Maurizi, Chao Gao, Filippo Berto

**Affiliations:** Department of Mechanical and Industrial Engineering, Norwegian University of Science and Technology (NTNU), 7491 Trondheim, Norway

**Keywords:** Engineering, Mechanical engineering, Mechanical properties, Computational science

## Abstract

Developing accurate yet fast computational tools to simulate complex physical phenomena is a long-standing problem. Recent advances in machine learning have revolutionized the way simulations are approached, shifting from a purely physics- to AI-based paradigm. Although impressive achievements have been reached, efficiently predicting complex physical phenomena in materials and structures remains a challenge. Here, we present an AI-based general framework, implemented through graph neural networks, able to learn complex mechanical behavior of materials from a few hundreds data. Harnessing the natural mesh-to-graph mapping, our deep learning model predicts deformation, stress, and strain fields in various material systems, like fiber and stratified composites, and lattice metamaterials. The model can capture complex nonlinear phenomena, from plasticity to buckling instability, seemingly learning physical relationships between the predicted physical fields. Owing to its flexibility, this graph-based framework aims at connecting materials’ microstructure, base materials’ properties, and boundary conditions to a physical response, opening new avenues towards graph-AI-based surrogate modeling.

## Introduction

In the ever-growing attempt to discover and design high-performing mechanical materials and structures, deformation, stress, and strain distributions are the essential information from which every other mechanical property or function can be deduced. With the recent explosion of additive manufacturing technologies, morphologically and physically sophisticated materials and structures with superior mechanical properties and functions, such as hierarchical composites^1–3^, geometrically interlocked structures^4–6^, and architected metamaterials^7–10^, can now be easily manufactured. Because of their geometric complexity^11^ and the intricate arrangement of constitutive materials with different mechanical properties^12^, predicting the physical response of such material systems with traditional methods, such as analytical models and numerical simulations, becomes easily intractable, especially when fast yet accurate screening of astronomically large datasets has to be carried out for materials discovery and design^13^. In addition, even traditionally manufacturable materials and structures involving highly nonlinear characteristics, such as hyperelasticity, plasticity, and post-buckling instability, require computationally expensive simulations, limiting materials research and discovery^14,15^. More generally, predicting deformation and stress fields of material and structural systems is a recurrent task in materials science and engineering and finding a fast yet accurate approach to it is an open challenging problem. Motivated by the limits of analytical models to efficiently predict the physical behavior of solid materials and structures, physics-based computational simulations, particularly finite element (FE) modeling, have so far represented the key factor to solve complex physical initial and boundary value problems, often involving highly nonlinear partial differential equations^16^. Emergence and growth of the machine learning (ML) field in the recent years has though demonstrated the possibility to outperform traditional numerical solvers, greatly speeding up simulations of physical systems^17–22^, from the use of physics-informed neural networks to extract velocity and pressure fields from flow visualization^23^ to the inverse-design of architected materials with extreme elastic properties using generative adversarial networks^24^. Given the importance of materials discover and design, linking materials’ micro- and meso- structure to mechanical properties (structure-to-property)^25–30^ and inverse-designing (i.e., given targeted properties, finding optimal designs) high-performing architected metamaterials^10,13,24,31–39^ have recently dominated the research scene. In both cases, materials performance is essentially dictated by local mechanical fields, such as stress and strain distributions, because of the effect of geometry, base materials’ behavior, and boundary conditions. Gaining advantage from the pixel-based convolutional neural networks, mechanical fields have been mainly studied on “digital” (i.e., discretized in the form of grids) material and structural systems^40–46^, as in^47^ where stress and strain fields were predicted on digital hierarchical composites, or in^48^ where heterogenous material microstructures were considered as images. One of the most popular and utilized numerical methods, such as FE modeling adopts mesh- instead of regular grid- representations to solve the underlying partial differential equations. With the intuitive extension of mesh information to graph representation, graph neural networks (GNNs)^49^ inherit all the advantages of using meshed domains. Furthermore, an efficient ML general framework capable of linking not only a material’s microstructure but also the constitutive materials’ properties (e.g., in a composite material) and boundary conditions to the physical response is still lacking.

Inspired by recent developments on GNNs for physical field predictions^50–52^, we propose a general method based on meshed geometries to predict stress, strain, and deformation fields in material and structural systems. The benefits of using deep GNNs instead of image-based models (such as convolutional neural networks) are potentially the following: (i) refined mesh close to stress/strain concentrators, like notches (i.e., defects) and material discontinuities, and curved smooth surfaces, allows for more accurate local predictions with less computational costs increase; (ii) unstructured mesh-based models allow to learn system’s behavior independently of resolution, meaning that different mesh size can be used at run time; (iii) Given their graph nature, architected truss metamaterials can more efficiently be represented by GNNs.

Here, harnessing the mesh-to-graph mapping, we propose a graph-based ML general method to predict deformed shapes, stress and strain fields in material and structural systems. To demonstrate the flexibility and generality of the proposed approach, we focus on three different material systems undergoing different complex mechanical phenomena, namely, plasticity of single-fiber composites, wrinkling of layer interfaces, and buckling instability of lattice metamaterials. We show that GNNs can learn loading conditions correlating deformed shape and stress field as well as physical relationships between material’s structure, and stress (strain) and deformation field. While image-based ML models, such as convolutional neural networks, variational autoencoders, and generative adversarial networks have been widely used to predict physical fields in hierarchical composites^53^, perforated structures^54^, additively manufactured microstructures with defects^45^, and heterogenous microstructures^42,48^, the current work presents a more flexible and general ML framework for the prediction of deformed shapes, stress and strain fields with GNNs.

**Figure 1:**
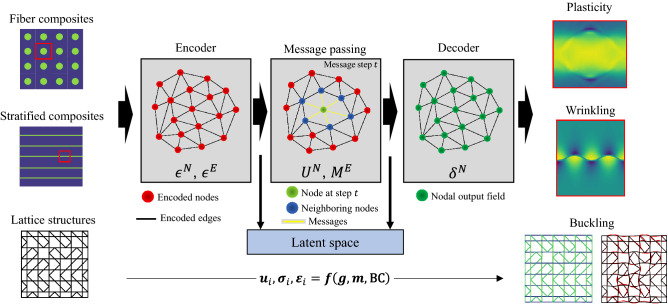
Schematics of the proposed ML model. The meshed material or structural system is first mapped onto a graph. Three examples of various complexity are here shown. Node and edge features are defined over the graph structure, carrying information of the system, such as node positions, node type (i.e., base material phase), or displacements on specific nodes. These features are first encoded into a larger latent space. Then, a message-passing module processes the graph features: each node acquires information from its neighboring nodes, learning relationships between different parts of the system. The transformed nodal quantities are finally decoded into output physical fields, here, deformation, stress, and strain fields ($u_i$, $\sigma _i$,$\varepsilon _i$). Providing geometric/topological information (${\textbf{g}}$), base materials’ behavior (${\textbf{m}}$), and boundary conditions ($\mathbf {\text {BC }}$), the model learns physical relationships with the considered fields. Once trained, the ML model can accurately predict physical fields of various material and structural systems subject to complex physical phenomena, such as wrinkling or buckling.

## Results

### Mesh to graph representation

Mesh domains used in FE modeling are collections of connected geometrical elements (such as lines, triangles, quadrilaterals, tetrahedra) defining solids, surfaces, or lines. The boundary value problem is often defined by the physical quantities of interest (such as displacement and stress) at specific locations on the boundary of the elements, namely, nodes (usually coincident with vertices). Considering the mesh only composed of nodes connected through edges, we identify mesh domains with computational graphs $G=(V,E)$, where *V* represents a set of *N* nodes connected to each other through *M* edges (*E*). The i-th node (in *V*) brings *n* features in the vector $v_i$ (such as nodal coordinates, and base material properties); similarly, the edge (in *E*) connecting the i-th and j-th node has a *m*-dimensional feature vector $e_{ij}$ (such as distance between nodes). As illustrated in Fig. 1, our model addresses the problem of predicting displacement ($u_i$), stress ($\sigma _i$), and strain ($\varepsilon _i$) fields (at the graph nodes) in material and structural systems, such as representative volume elements (RVEs) and finite-size lattice structures. Given the flexibility of the graph representation, all the factors that determine the material’s mechanical response, i.e., geometry/topology (${\textbf{g}}$), base materials behavior (${\textbf{m}}$), and boundary conditions ($\mathbf {\text {BC }}$), can be encoded into the node and edge features, and graph connectivity (as next shown for three material systems).

### Model description

GNNs is a class of deep neural networks that operate directly on graph data (i.e., non-Euclidean data), instead of vectorized or image-data^55^. In our work, we develop an encoder-decoder ML model to approximate the relationship (*f* in Fig. 1) between material and structural characteristics and conditions, and physical fields (displacement, stress, and strain). Our model consists of three components, the Encoder, the Message Passing, and the Decoder (Fig. 1). From the graph representation of the material system, the Encoder encodes the node and edge features into a latent space (of larger dimension *d*) using the neural networks $\epsilon ^N$ and $\epsilon ^E$ at each node and edge, respectively. These encoded features are processed by the Message Passing module, which aggregates first information from the neighborhood of each node (through the neural network $M^E$), and then updates the node state using the neural network $U^N$; these two operations represent a message pass. After *L* message passes, the latent node features are transformed by the neural network $\delta ^N$ (i.e., the Decoder) into the field outputs. The model is trained by supervising on nodal physical quantities (i.e., $u_i$, $\sigma _i$, $\varepsilon _i$) obtained by FE simulations (ground truth). Overall, the GNN model takes a graph representing the material system as input, and outputs physical fields. As measures to evaluate the model performance, we use the error map, defined as the nodal difference between the ground truth and predictions, and the mean absolute error, MAE. Moreover, to measure the ability of the model of predicting derivative material properties, we use the mean relative error for the constitutive stress-strain response of the whole RVE (when the stress field evolution is studied). More details are provided in Methods, and Supplementary Materials.

Our trained ML model can predict complex mechanical phenomena, such as wrinkling of thin interfacial layers and buckling instability of lattice materials, linking the geometry/topology, the base material properties, and the loading conditions to the deformation, stress and strain fields. Learning physical relationships between deformation and stress (or strain) fields from data, the model predicts realistic global mechanical behavior even when input information is not enough to capture the actual local phenomenon (e.g., the eigen-modes are not provided as input for post-buckling predictions). Indeed, attempting to minimize the global MAE, the average behavior is more easily captured by the model in the absence of data related to the considered physical phenomenon (next evidence is provided). To demonstrate the power of deep GNNs to learn and predict the physics of complex mechanical phenomena in different classes of materials and structures, in the following sections we report three different problems solved by our ML model in order of increasing complexity.

### Learning a class of composite materials

**Figure 2:**
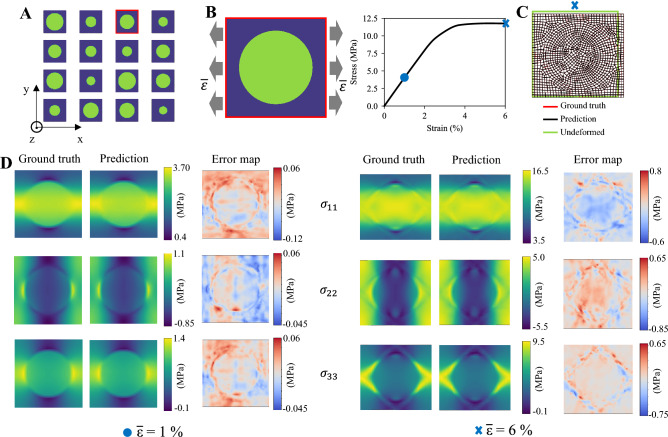
Predicting elasticity and plasticity in transversally loaded unidirectional fiber composites. (**A**) Examples of RVEs from the dataset having different fiber volume fraction. (**B**) A typical RVE ($f_v$=37.55 $\%$) subject to macroscopic uniaxial tension strain (${\overline{\varepsilon }}$) together with the corresponding macroscopic stress-strain curve. The symbols in the plot indicate small (${\overline{\varepsilon }}$=1 $\%$) and large (${\overline{\varepsilon }}$=6 $\%$) deformations, corresponding to linear elasticity and plasticity, respectively. (**C**) Comparison of the FE simulated (i.e., ground truth) and ML predicted deformed mesh for ${\overline{\varepsilon }}$=6 $\%$. (**D**) Comparison of the ML predicted and FE simulated stress fields in the RVE shown in (**B**), randomly chosen from the test dataset, for small and large deformations, with the corresponding error map (i.e., difference between prediction and ground truth). The stress fields are plotted over the corresponding deformed shapes (exact relative scale between small and large deformations), while the error maps are shown in the undeformed configuration. Analogous results for the shear stress field ($\sigma _{12}$) are reported in Supplementary Materials.

Here, we focus on traditional unidirectional fiber composite materials subjected to plain strain uniaxial tensile loading. For simplicity of conveying the key ideas of our method, a grid-like periodic fiber distribution is considered by analyzing a single-fiber RVE (representing the microstructure), as shown in Fig. 2A,B. Two constituent materials compose the material microstructure, the hard phase (i.e., fiber) and soft phase (i.e., matrix). The fiber has linear elasticity, whereas the matrix has elasto-plastic (J2 plasticity) behavior. To simulate high-contrast material discontinuity, in terms of Young’s modulus, the hard phase is 10 times stiffer than the soft phase (see Methods). Fully characterizing the microstructure’s geometry, the volume fraction of the fiber, $f_v$ is employed as the only independent geometric parameter *g*; for each value of $f_v$, it is associated a unique fiber’s radius (Fig. 2A shows different microstructures). Linearly sampling $f_v$ in the range 0.05–0.5, a dataset of 500 microstructures is generated, then split into 90 $\%$ of training data and 10 $\%$ of test data (sensitivity analysis with training data density reported in Fig. S12). Imposing periodic boundary conditions (PBCs) on the boundaries of each RVE (see Methods), the displacement $u_i=(u_i^1,u_i^2)$, and stress $\sigma _i= (\sigma _i^{11},\sigma _i^{22},\sigma _i^{33},\sigma _i^{12})$ fields obtained from FE simulations are regarded as the ground truth for the ML model’s output. The model’s input geometric information *g* of the microstructure is encoded into the graph topology (through the node connectivity), and the node and edge features. Specifically, undeformed nodal coordinates $x_i$ and node type $\xi _i$ (equals 0 for matrix, 1 for fiber) are encoded into the node features, $v_i$. Relative distance between the i-th and j-th node $x_{ij}=x_i-x_j$ in the undeformed configuration and its absolute value $|x_{ij}|$ are encoded into the edge features, $e_{ij}$.

A typical RVE with the corresponding macroscopic stress-strain curve from the test dataset is shown in Fig. 2B. To demonstrate the ML model’s capability of capturing the small and finite deformation and stress field in fiber composites, in Fig. 2C-D we report the comparison between the FE simulations (ground truth) and model predictions for two macroscopic applied strain values, ${\overline{\varepsilon }}$= 1 and 6 $\%$ (model trained separately). An average MAE of $\sim$ 0.02 and 0.04 is obtained on the test data (i.e., 50 microstructures) for the elastic and plastic regime, respectively. The deformation of the microstructure is accurately predicted by our ML model, as depicted in Fig. 2C for the plastic regime. Analogously, the ML predicted stress components $\sigma _{11},\sigma _{22},\sigma _{33}$ distributions, shown in Fig. 2D, greatly resemble the numerical simulations for both strain regimes. The accuracy of the predictions is further confirmed by the error map, which additionally identifies the small local regions with larger errors (mostly close to the fiber-matrix boundary of high stress and strain gradient, as in^48^), contributing to accuracy drops. Remarkably, also complex stress patterns, as for the shear stress, $\sigma _{12}$, with low amplitude (compared to the main component $\sigma _{11}$) are captured by our predictions, as shown in Fig. S2. Deformation and stress field comparisons for other RVEs in the test dataset are reported in Fig. S3 and S4. While often equivalent stress distributions, such as von Mises stress, are adopted as single output^45,48^, our model learns not only the whole stress tensor (i.e., multiple components) field but also the corresponding deformed shape (Fig. 2C). Learning the mechanics of microstructures with different fiber’s radii (thus, volume fraction), the proposed model can thus accurately predict the small and finite deformation, and stress field in both dilute and dense fiber-reinforced composites.

### Deformation and stress field evolution

In the earlier results on fiber composite microstructures, the ML model was trained separately for different levels of macroscopic applied strain. Here, we investigate whether our model can simultaneously learn multiple loading steps at different magnitudes i.e., the evolution of deformation and stress field. To demonstrate the ability of the model to predict large local deformations, we first consider the same previous dataset but subject to displacement boundary conditions (instead of PBCs). In this way, harnessing the graph structure of the mesh to inform the ML model on the boundary conditions, a vector, $u_i^{\mathbf {\text {BC }}}=(u_i^{(1,\mathbf {\text {BC }})},0)$ representing the applied displacement on the i-th node, is introduced as additional node feature (i.e., $\mathbf {\text {BC }}$ in Fig. 1) . To further reduce the computational cost for training data generation and model training, a coarser mesh and linear elasticity are here adopted, and only 100 total data are considered. Without the hypothesis of plasticity, the macroscopic stress-strain responses are linear (less complex); this hence reduces the amount of required training data. Five loading steps are linearly sampled in the range 1–8 $\%$ of effective applied strain (ratio between the applied displacement and the RVE’ size) for each microstructure, treating the temporal variable (i.e., loading steps) as a parametrization of the data distribution i.e., a sequence of graphs. In addition, to make the approach more general (for future applications on path-dependent problems), we insert two recurrent layers after the message-passing module, interpreting the latent node features as hidden states (Methods), finally transformed to output fields by the decoder (Fig. 1). An average MAE of $\sim$ 0.07 is obtained on the test data (average over all the microstructures and steps) and a remarkable resemblance between the ML predictions and FE simulations is achieved, as shown in Fig. S5 and Movie S1. The large transversal (to the loading direction) deformations of the microstructure are captured by our model, confirming that GNNs can predict smooth surface deformations with accuracy close to that of high-fidelity FE solvers.

Moreover, to test the capability of the model to predict physical responses on unseen applied strains , five additional loading steps are sampled in the applied strain range (1–8 %), with a total of ten applied strain levels. The model, trained only on five steps, is tested against all the ten strains. The result is shown in Movie S2, where the $\sigma _{11}$ stress component is displayed; an average MAE of $\sim$ 0.07 is obtained, as before. Analogous results, not shown here for the sake of brevity, hold by sampling the strain range by an arbitrary number of loading steps (we tested up to 25), and training the model only on a few of them. The model can thus accurately predict the selected physical fields on different loading steps from the five training ones (spanning all the applied strain range). This approach may be helpful to reduce the amount of required training data for fields evolution prediction. Indeed, by training the model on a few time steps, it would be able to predict the fields evolution also for unseen strain levels in between.

**Figure 3:**
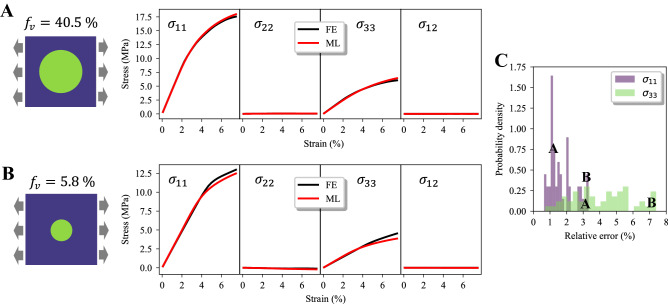
Derivative macroscopic material response from the GNN-predicted stress field evolution of unidirectional fiber composites. Stress–strain curves derived from ML-predicted and FE-simulated fields for $f_v = 40.5$ % (**A**), and $f_v = 5.8$ % (**B**). (**C**) Mean relative error distributions evaluated on the test dataset for the two non-zero macroscopic stress components. The letters in (**C**) refer to the relative errors of the individual non-zero stress components of the corresponding RVEs in (**A**) and (**B**). The strain on the x-axis in (**A**) and (**B**) is the macroscopic applied strain (${\overline{\varepsilon }}$). For more details on stress-strain curves derivation see Methods.

To further demonstrate that our model can accurately predict derivative material properties from more complex stress fields, linear hardening plasticity is here introduced for the matrix of the composite together with PBCs (more details in Methods). These latter are represented as additional node features, $\varepsilon _i^{\mathbf {\text {BC }}}$ being the applied macroscopic strain component (**BC** in Fig. 1). The model is trained on ten loading steps and it is tested on 25 levels of strains in the same range (0–8 %). In Fig. 3 the predicted and simulated stress-strain responses of two RVEs with different fiber volume fractions as well as the mean relative error distributions on the test dataset are reported for the two non-zero macroscopic stress components (average over the RVE). Although higher average MAE values ($\sim$ 0.10) for the physical fields are obtained, the macroscopic predictions are virtually indistinguishable from the simulated responses, exhibiting maximum relative errors below 3.5 and 7.5 % for the components $\sigma _{11}$ amd $\sigma _{33}$, respectively (Fig. 3). In addition, without inputting the information of plain strain uniaxial tension into the GNN model, the predicted macroscopic stresses are consistent with such applied condition. Accordingly, the model predicts the stress components $\sigma _{22}$ and $\sigma _{12}$ to be zero, with a scatter below 1 % of the peak of the dominant component, $\sigma _{11}$.

Overall, the performance of our GNN models on the two datasets, comprising only elastic phases with larger deformations or more complex linear hardening plasticity, seem to be promising. However, we observe that the mean errors of field evolution ($\sim$ 0.07–0.10) are higher than those of separate predictions ($\sim$ 0.02–0.04), i.e., model trained on a specific macroscopic strain. For the elastic case, only 90 microstructures (90:10 training-test data ratio) each with five loading steps are employed as training data, resulting in a really small dataset, thus, limiting the maximum accuracy. For the plastic case, 450 microstructures each with ten loading steps are used; however, the additional complexity of linear hardening plasticity (characterizing the matrix), introduces a path-dependent physics, calling for more data. To understand the trade-off between the predictive performance and training costs, we report a sensitivity analysis in Fig. S12, showing that not only the average MAE but also the scatter decreases with larger training datasets. Owing to the large graphs involved (mesh-to-graph mapping), future works may thus focus on ways to reduce such graph size, providing a way to exploit larger training datasets.

### Wrinkling of interfacial layers

**Figure 4:**
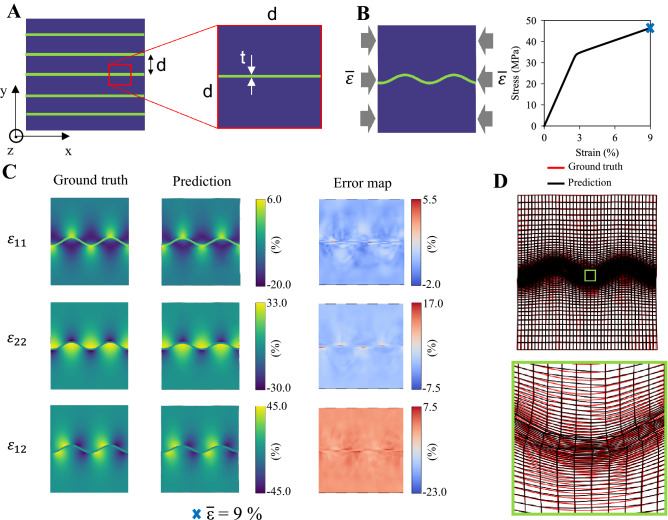
Predicting wrinkling of interfacial layers in stratified composites. (**A**) Schematic of a stratified composite composed of thin hard layers immersed in a soft matrix. Both phases have linear elasticity. The inset shows an arbitrary RVE of size *d*, corresponding to the distance between layers, and layer’s thickness *t*. Here, the two phases’ material properties (*E*,$\nu$) are varied instead of the geometric parameters. (**B**) Wrinkled interface under macroscopic uniaxial compression (${\overline{\varepsilon }}$) together with the corresponding macroscopic stress-strain curve. The symbol identifies the post-wrinkling regime for ${\overline{\varepsilon }}$=9 $\%$. This configuration is characterized by a Young’s moduli ratio $E_1$ / $E_0$=741 and matrix’ Poisson’s ratio $\nu _0$=0.13. (**C**) Comparison of the ML predicted and FE simulated strain fields in the interfacial layer shown in (**B**), randomly sampled from the test dataset, for ${\overline{\varepsilon }}$=9 $\%$, with the corresponding error maps. The stress fields are plotted over the corresponding deformed shapes, while the error maps are shown in the undeformed configuration. The strain component $\varepsilon _{33}$ is overall zero because of the plain strain hypothesis, and it is thus not here reported. (**D**) Comparison of the ML predicted and FE simulated deformed mesh of the configuration in (**B**) for ${\overline{\varepsilon }}=9 \%$.

Although stress and strain fields in material systems with variable base material composition exhibiting complex behavior could reasonably be predicted by pixel-based ML models using high resolution images^45,47,48^, accurately predicting also complex deformed shapes would be computationally costly. To face this challenge using our GNN model, as an example we focus here on the formation of wrinkled interfaces (i.e., instability) in soft layered composites^56^. The stratified composite (Fig. 4A) is composed of thin hard interfacial layers of thickness *t*, periodically arranged at distance *d* and embedded in a soft matrix; both phases have linear elasticity, with Young’s modulus $E_0$ and $E_1$ for the soft and hard phase, respectively. Assuming periodic geometric pattern, we consider an RVE of size *d* (inset of Fig. 4A), subjected to plain strain uniaxial compression with applied macroscopic strain ${\overline{\varepsilon }}$= 9 $\%$ under PBCs (see Methods). As already known, the layer instability is governed by the distance-to-thickness ratio, *d*/*t*, Young’s moduli ratio $E_1$/$E_0$ and matrix’ Poisson’s ratio $\nu _0$^56^. To verify whether our model can learn a relation between the base materials properties $m=(E,\nu )$, and displacement and strain fields $(u_i, \varepsilon _i)$, we keep the geometric parameter *g* = *d*/*t* constant (avoiding long-wave instability^56^), while varying $E_1$/$E_0$ and $\nu _0$ in the range 50–1000 and 0.01–0.49, respectively. Linearly sampling 100 values for $E_1$/$E_0$, and 5 values for $\nu _0$, a dataset of 500 configurations (i.e., total combinations) is then constructed, and split into 90 $\%$ of training data and 10 $\%$ of test data (sensitivity analysis with training data density reported in Fig. S12). The base materials information is naturally encoded into the node features, substituting the previously used node type $\xi _i$ with *m* for each node.

The compressed interfacial layer tends to deform into a wavy pattern upon the onset of buckling instability, as shown in Fig. 4B. For different layer and matrix properties, various wavy deformed shapes and strain field amplitudes are exhibited (see Fig. S6). Here, the most challenging task is capturing the complex wavy patterns (i.e., amplitude and wavelength of the deformed shape), and strain contours (i.e., spatial distribution and local amplitude) upon buckling of the interfacial layer for different base material properties. Indeed, as the phenomenon becomes more complex and larger deformations occur, the ML model struggles to have similar low MAE values on the test dataset as those of the previous example (fiber composites). Nonetheless, a non-uniform distribution of MAE values (both for training and test dataset) is exhibited (Fig. S7). Most of the predictions exhibit low MAE, as shown in Fig. 4C, closely resembling the FE simulated wavy patterns as well as the strain distributions. The average MAE value (on the test dataset) of $\sim 0.22$ can be explained by highly localized mismatches in the strain field amplitude in a few regions close to the interface (error map in Fig. 4C), and in the wavy deformed shape for some test configurations (Figs. S7–S8). Specifically, almost independently of $\nu _0$, the MAE of the predictions on the whole dataset tends to be smaller for microstructures with higher $E_1$/$E_0$, corresponding to long-wavelength, high-amplitude wavy patterns (Fig. S7). These results suggest that complexity rather than largeness of the deformation limits the accuracy of predictions. Despite predicting the wrong wavy pattern (i.e., wavelength) for low stiffness ratios, the trained ML model tends to learn a relationship between the curvature of the interfacial layer and the strain distribution (Fig. S8). For each strain component, positive and negative strains are associated to the concavity of the wavy pattern (Fig. S8). Considering for example the components $\varepsilon _{11}$ and $\varepsilon _{22}$ in Fig. S8, although the predicted wavelength does not match the real one, the tensile and compressive regions in the composite matrix are qualitatively well captured based on the layer concavity. To better evaluate our predictions, in Fig. 4D we compare the FE simulated and ML predicted wrinkled meshed interfaces. Although deviations of the predicted deformation from the smooth numerical solution exist (inset in Fig. 4D), the two meshes globally overlap, indicating that the amplitude and wavelength of the post-buckled shape of the layer are accurately predicted by the ML model. This example demonstrates that GNNs, which allow resolution increase close to regions where the local phenomenon is expected to occur, can accurately capture localized complex phenomena, just in a purely data-driven supervised setting. For future research, we argue that smoothness in the ML predicted solution might be enforced by introducing physical constraints into the GNN model, increasing accuracy and generalizability. To investigate if an image-based ML model can similarly solve this problem using the same dataset, we implement a U-Net (recently adopted for heterogeneous materials with variable base material properties^48^) and report the results in Fig. S9. After several empirical tests performed by varying the perceptive field (increasing the kernel size, the dilation step, and network’s depth), the U-Net model tends to predict uniform strain fields (on the three components) with a relatively low average MAE of $\sim$ 0.07 yet without capturing any local deformation. These results suggest that a GNN framework can not only predict highly localized phenomena for composites with variable base material properties but also reduce the need of training data compared to image-based ML models.

### Compression of architected metamaterials

**Figure 5:**
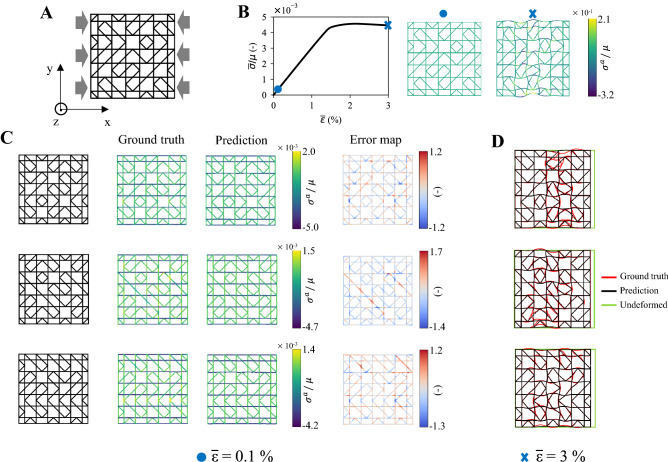
Predicting compressive behavior of lattice metamaterials. (**A**) Representative lattice structure under compressive loading. (**B**) Normalized effective stress-strain curve of the structure in (**A**) together with the normalized FE simulated stress field (i.e., axial stress, $\sigma ^a$, on the lattice’s beams) for small (${\overline{\varepsilon }}=0.1 \%$) and large (${\overline{\varepsilon }}=3 \%$) deformations. (**C**) Comparison of the ML predicted and FE simulated stress field for small deformations (${\overline{\varepsilon }}=0.1 \%$) in three randomly chosen geometries from the test dataset, together with the corresponding error map. (**D**) ML predicted and FE simulated post-buckled deformed shapes for ${\overline{\varepsilon }}$=3 $\%$. The base material has incompressible nonlinear elasticity (hyperelasticity) with initial shear modulus $\mu =14.5 \,\hbox {MPa}$.

While image representations are generally effective to represent fully dense material systems, such as those previously analyzed, they are not when dealing with architected metamaterials with low volume fraction, such as lattice structures, in which the solid phase is sparsely distributed. These structures are instead more naturally suitable for graph representation. As an example, here we exploit our GNN model to predict the deformation and stress field in finite-size lattice structures under compressive loading (Fig. 5A,B). Based on a bottom-up generation procedure featured in our previous work^57^, a dataset of 762 structures is constructed with 2 $\times$ 2 tessellations of randomly generated unit cells (Supplementary Materials) and split as before in training and test dataset (sensitivity analysis with training data density reported in Fig. S12). The lattice structures are composed of incompressible hyperelastic beams with initial shear modulus $\mu$ and uniform thickness *t*, and have constant volume fraction ${\overline{\rho }}$. Separately trained on two different deformation regimes i.e., small and large deformations, the ML model is characterized by different node and edge features, and output. For small deformations, only the nodal coordinates of the undeformed mesh (10 beam-elements per beam) are encoded into the node features, and analogous edge features as before are here adopted; the output is represented by the displacement field $u_i$, and axial stress $\sigma _i= \sigma _i^a$ along the beams. For large deformations, accounting for local buckling, the critical eigen-mode coordinates, $\tilde{x_i}$ are additionally encoded into the node features, and the corresponding node-to-node distances ($\tilde{x_{ij}}$ and $|\tilde{x_{ij}}|$) are included into the edge features; only the displacement field is output by the model. More details are provided in Methods, and Supplementary Materials.

To demonstrate our ML model can learn the compressive behavior of non-uniform lattice structures for small and large deformations, in Fig. 5C,D we report the FE simulated and ML predicted stress distribution $\sigma _i^a$ for small effective strain (${\overline{\varepsilon }}=0.1 \%$), and deformed shapes after the onset of buckling instability (${\overline{\varepsilon }}=3 \%$), for three different architectures randomly sampled from the test dataset (see Fig. S10 for other lattices). In the elastic regime, the stress field is overall captured by our model with MAE values $\sim 0.29$ (Fig. 5C). Carrying most of the load through the beams aligned with the loading direction, the lattice structures are mainly stressed along the horizontal direction (*x*-direction). Predicting this behavior thus suggests that the ML model effectively learns the mechanics of the structure under compressive loading. The error map in Fig. 5C confirms these results, except for a few localized regions, mainly represented by the lattice junctions, where the stress concentrations tend to be smoothed by the ML model to reduce the overall loss. This limitation, however, does not impact the prediction of the overall mechanical behavior of the structure without considering local cracking and damage mechanisms, beyond the scope of this example. Upon reached the critical applied displacement, the structure locally buckles and localizes the deformation transversally to the loading direction (Fig. 5B). The global displacement (thus, the stiffness) of the structure is captured by the ML model, as shown in Fig. 5D. Despite the highly localized nonlinear deformation, converging to MAE values $\sim 0.39$ on the test dataset, the ML model can also predict the complex post-buckled deformed shape with satisfactory approximation (Fig. 5D). The mismatch between the predicted and simulated beams’ local deformations together with the good approximation of the global structure displacement suggests that the model, trying to minimize the overall error, gets stuck in a local minimum during training. We expect that much larger training datasets and physics constraints in the model could reduce such local mismatch. We also notice that without using the critical eigen-mode information as input, the model is prone to converge to the un-buckled deformed configuration, while properly predicting the global displacement (Fig. S11). This observation suggests that the GNN model tends to learn actual physical relationships between the given inputs and outputs (Fig. 1).

## Discussion

Here, we have proposed a mesh-based ML approach for the prediction of deformation, stress and strain fields in material and structural systems exhibiting complex physical phenomena using GNNs. The neural networks are trained on a few hundred simulation data yet accurately predict complex phenomena, such as wrinkling of interfacial layers and buckling instability of architected metamaterials, hence, showing high versatility and broad applicability. Harnessing the natural mesh-to-graph mapping and the expressive power of GNNs, our model learns the physical relationships between geometry and topology, constituent materials properties, boundary conditions, and mechanical fields in various classes of materials and structures, from fiber composites to architected lattice structures (Fig. 1). With the ambitious goal of surrogating or complementing FE simulations of mechanical systems, the proposed model, once trained, dramatically reduces the computation time from minutes, hours, or days (typical for FE solvers) to fractions of a second (see Table S3). In addition, while the training process represents the computational bottleneck, once trained, GNNs can quickly predict mechanical fields in the specific class of materials and structures where have been trained independently of the complexity of the problem.

Nevertheless, limits related to (1) the graph structure of GNNs, and (2) the purely supervised data-driven set-up here used, exist. Regarding (1), GNNs are memory-demanding during training; therefore, as the number of nodes in the mesh rises, the training phase becomes increasingly more expensive. In addition, a trade-off between mesh refinement and far-field phenomena appears: the more accurately local fields are predicted via mesh refinement, the more message passing steps (computational cost) are needed to accurately capture far-field phenomena (i.e., information spatially far away from the mesh refinement). Regarding (2), although our model can predict complex phenomena with a small amount of training data (a few hundreds), it still lacks of generalizability (e.g., predicting “unseen” boundary conditions) as well as the accuracy is still not completely comparable to that of high-fidelity numerical solvers. For future research, we guess that by constraining the GNN solution to physics laws may largely improve the accuracy^22^, at the cost of higher training time. Despite these trade-offs are not negligible, the proposed graph-based ML framework represents a first step towards more powerful surrogate models, especially suitable for cellular solids, as architected truss metamaterials. To evaluate the applicability of the model, we additionally report in Fig. S12 a sensitivity analysis of the model performance with the training data density. For the three mechanics problems, we show that although the average accuracy of field predictions does not quickly rise, the scatter considerably decreases with the training density.

As a proof of concept, we have additionally demonstrated that our approach can be extended to predicting mechanical fields in material systems under multiple loading steps (e.g., for different applied strains). Combining GNNs with recurrent neural networks in a dynamic-graph framework^55^ to predict physical fields for different applied external excitations (such as macroscopic strain in RVEs) could represent a promising tool for exhaustively modeling nonlinear and path-dependent phenomena in materials, such as nonlinear elasticity and plasticity. Carrying all the needed information, the variable physical fields would provide the macroscopic material response (e.g., stress–strain curve) as shown in Fig. 3, from which material properties, such as strength and toughness, could be easily extracted. In addition, stemming from the flexibility of graph representation and the expressive power of GNNs, mixed loading conditions may be easily encoded into the model through the node or global graph features^55^ (e.g., different displacement components applied to boundary nodes), and topology optimization may be integrated with the proposed model to address problems related to curves and surfaces’ smoothness. This work not only provides a novel method to predict complex physical phenomena using graph-based ML models but also opens new avenues to designing advanced materials like mechanical or functional truss metamaterials.

## Methods

### FE modeling

The datasets are generated by FE modeling, using the commercial software Abaqus/Standard (Dassault Systemes Simulia Corp., 2017), considering the displacement, stress and strain fields as the ground truth for comparison with the ML results. All simulations are carried out in 2D under static loading. Automatic time step procedure (i.e., increment size in Abaqus) is adopted, except for multi-step predictions (i.e., evolution of physical fields) where a fixed time step of 0.01 is set; the static loading is overall divided in 101 steps. The logarithmic strain (LE in Abaqus) is employed as strain measure for the simulations involving large deformations. For the fiber and stratified composites, plain strain elements (“CPE4R” in Abaqus) are used with a global mesh size of 0.03 and locally refined mesh with 40-by-10 elements (*x* and *y* direction in Fig. 4A) on the interfacial layer, respectively. Timoshenko-beam elements (B22 in Abaqus) are used to mesh the lattice structures, with a global mesh size of 10 elements per physical beam. To obtain stable results, convergence analysis is performed for the three material systems. All the computations are performed on a Intel Xeon E3-1270, 3.60 GHz, CPU core.

### Datasets

#### Unidirectional fiber composites

The first dataset is composed of 500 RVEs characterized by different fiber volume fraction (i.e., fiber’s radius), linearly sampled in the range 0.05–0.5. The RVE’s shape is a square with size arbitrarily set to 1 mm. Applying a macroscopic plain strain tensile loading strain along the *x*-direction (Fig. 2A) of 6 $\%$ to each RVE subjected to PBCs, the nodal displacement, $u_i=(u_i^1,u_i^2)$, and stress, $\sigma _i= (\sigma _i^{11},\sigma _i^{22},\sigma _i^{33},\sigma _i^{12})$ fields at two loading steps (1 and 6 $\%$ of strain) are collected in the dataset as the ground truth. The matrix is modeled as an elasto-plastic (J2-perfect-plasticity) solid with Young’s modulus of 200 MPa, Poisson’s ratio of 0.3, and yield strength of 10 MPa. Representing the hard phase, a linear elastic model is instead adopted for the fiber, with Young’s modulus of 2000 MPa and Poisson’s ratio of 0.3. For the multi-step predictions, two datasets are generated. For the first, composed of 100 RVEs, displacement boundary conditions are imposed (instead of PBCs) up to 8 $\%$ of effective strain (ratio between the applied displacement and the RVE’ size). A linear elastic model is adopted for both phases with same previous parameters. For the second, composed of 500 RVEs, PBCs are imposed up to 8 % of macroscopic applied strain. Same previous base material properties are adopted, except for the matrix which is modeled using a linear hardening plasticity with a tangent modulus $E_y = E \, \text {/} \, 3$. The macroscopic strain-strain curves shown in Fig. 3 are derived by averaging the local stress field for each applied macroscopic strain value. More details on PBCs can be found in Supplementary Materials.

#### Wrinkled interfacial layers

The dataset includes 500 different combinations of base material properties. The two phases have linear elastic response. The soft phase (i.e., the matrix) has constant Young’s modulus $E_0=200 \,\text {MPa}$, and variable Poisson’s ratio $\nu _0$ in the range 0.01–0.49. The hard phase (i.e., the interfacial layer) has variable Young’s modulus $E_1$ in the range $10^4$–$2 \times 10^5\,\hbox {MPa}$, and constant Poisson’s ratio $\nu _1=0.3$. Linearly sampling 100 values for $E_1$, and 5 values for $\nu _0$, 500 combinations are obtained. To limit the data generation, the geometric parameter $g=d$/*t* is set to 50, arbitrarily assuming $d=1 \,\text {mm}$. We simulate nonlinear post-buckling behavior of the interfacial layer (i.e., wrinkling) by (1) conducting eigenvalue analysis, (2) applying the critical eigen-mode to the RVE as imperfection, and (3) carrying out a nonlinear static analysis with large deformations. Applying a macroscopic compressive loading strain along the *x*-direction (Fig. 2A) of 9 $\%$ to each RVE subjected to PBCs, the nodal displacement, $u_i=(u_i^1,u_i^2)$, and strain, $\varepsilon _i= (\varepsilon _i^{11},\varepsilon _i^{22},\varepsilon _i^{33},\varepsilon _i^{12})$ fields are collected in the dataset as the ground truth. More details on PBCs and wrinkling can be found in Supplementary Materials.

#### Lattice structures

The dataset is composed of 762 finite-size $2 \times 2$ tessellations of randomly generated unit cells (see Supplementary Materials) with constant relative density ${\overline{\rho }}=20 \%$. The lattice beams are characterized by a rectangular cross section with in-plane thickness $t= 0.14 \,\hbox {mm}$ and depth $H=10 \,\hbox {mm}$. To reduce the boundary effects, a thicker frame around the structures is considered with $t= 0.30 \,\hbox {mm}$. An incompressible hyperelastic Neo-Hookean material model with initial shear modulus $\mu =14.5 \,\text {MPa}$ is adopted to model the lattice beams. Uniaxial compressive displacement (along the *x*-direction, Fig. 5A) is applied to the right edge, while constraining the left side. To account for buckling instability, we simulate nonlinear post-buckling behavior of the structures by (1) conducting eigenvalue analysis, (2) applying the critical eigen-mode to the structure as imperfection, and (3) carrying out a nonlinear static analysis with large deformations and material nonlinearities. The nodal displacement, $u_i=(u_i^1,u_i^2)$ and stress, $\sigma _i= \sigma _i^a$ fields for two deformation regimes i.e., effective strain of 0.1 and 3 $\%$ before and after buckling, respectively, are then collected in the dataset as the ground truth. More details on the unit cell generation can be found in Supplementary Materials.

### Structure of the ML model

The ML model is implemented using PyTorch Geometric^58^ in the PyTorch framework^59^. The model consists of an encoder, a message-passing module, and a decoder. The encoder function is encoding node, $v_i$, and edge, $e_{ij}$ features into a larger latent space. This task is carried out using two neural networks, $\epsilon ^N$ and $\epsilon ^E$ for the node and edge features, respectively. Each feature is input into the corresponding network, composed of two layers of width *d* (namely, latent size), each associated with a ReLU nonlinear activation function, and finally followed by a LayerNorm layer, which performs element-wise Layer Normalization^60^ using mean and standard deviation over a mini-batch. The resulting latent graph is then processed by the message-passing module. For each node, the latent neighboring node and edge features are transformed by the neural network, $M^E$, and aggregated via summation; this represents the message passed by the neighborhood of one node. The message together with the previous node features (namely, node state) is then updated by the neural network, $U^N$, generating a new node state. Before aggregation, the message represents the new edge features. The new node states and edge features are summed to the corresponding previous states (i.e., sum of residuals). This process is repeated *L* times (i.e., message steps). The networks $M^E$ and $U^N$ have the same architecture of the networks in the encoder. After *L* message steps, in the decoder the latent node states are transformed by the neural network, $\delta ^N$, into field outputs. For multi-step predictions, to make the approach more general, two gated recurrent units (GRUs) are inserted after the message-passing module, interpreting the latent node states as hidden states (see Supplementary Materials). The node and edge features of each sample in the dataset are normalized using the function StandardScaler in the sklearn Python library. Analogously, the ground truth (i.e., nodal physical fields) is normalized using the mean and standard deviation computed on the training data by an in-house function; the ML model outputs are then de-normalized for performance evaluation and visualization. For training our model, we use the mean absolute error, MAE, as loss function, and the Adam optimizer, setting the initial learning rate to 0.01 with exponential decay by $\gamma =0.9$ every epoch. To reduce overfitting, an L2 regularization technique with weight decay of $5 \times 10^{-4}$ is adopted in the Adam optimizer. In addition, to reduce the memory consumption a mini-batch technique is employed during training. Each dataset is split into 90 $\%$ of training data and 10 $\%$ of test data. In Table S1 we report the specific values of latent size, message steps, batch size, and training epochs for each dataset. For the sake of clarity, in Table S2 the node and edge features, and the output fields for each dataset are additionally reported. Without loss of generality, optimization of the networks’ architecture and hyperparameter sensitivity analysis are not here performed, being outside the goal of this work. All the ML training and inference is performed on a GPU NVIDIA Quadro P2000, 5GB (dedicated memory); an Intel Xeon E3-1270, 3.60 GHz, CPU core is also utilized for inference (predictions on unseen data) to have a fair comparison with FE simulations. More details can be found in Supplementary Materials.

## Supplementary Information

Supplementary Information 1.

Supplementary Information 2.

Supplementary Information 3.

## Data Availability

The datasets used and/or analyzed during the current study are available from the corresponding author on reasonable request. The code of the ML models is available on GitHub https://github.com/marcomau06/GNNs_fields_prediction.
