# Supplementary material for: Predicting stress, strain and deformation fields in materials and structures with graph neural networks
Source: Sci Rep. 2022 Dec 17;12:21834. doi: 10.1038/s41598-022-26424-3 (PMC9759553; doi:10.1038/s41598-022-26424-3)
Supplement: Supplementary file 1 — Supplementary Information 1. [file 41598_2022_26424_MOESM1_ESM.pdf]

Supplementary Materials for

Predicting stress, strain and deformation fields in materials and structures with graph neural networks

Marco Maurizi*, Chao Gao, Filippo Berto

*Corresponding author. Email: marco.maurizi@ntnu.no.

This file includes:

Supplementary Text
Figs. S1 to S12
Tables S1 to S3
Legends for movie S1 to S2
Supplementary references

Other Supplementary Materials for this manuscript include the following:

Movie S1
Movie S2

Supplementary Text

Details of the FE modeling

Periodic boundary conditions (PBCs)

To analyze the influence of micro- and meso-structure of material systems on their macroscopic mechanical behavior, the application of periodic boundary conditions (PBCs) on representative volume elements (RVEs) is needed. In this work, unidirectional fiber composite and stratified composite RVEs are subject to a 2D macroscopic deformation gradient $\overline{\boldsymbol{F}}$ under PBCs by enforcing:

$$\boldsymbol{u}_{A_k} - \boldsymbol{u}_{B_k} = \left(\overline{\boldsymbol{F}} - \boldsymbol{I}\right)\left(\boldsymbol{X}_{A_k} - \boldsymbol{X}_{B_k}\right), \quad k = 1, \dots, K$$

where $\boldsymbol{u}_{A_k}$ and $\boldsymbol{u}_{B_k}$ are displacements of K pair of points periodically located on two opposite boundaries of the RVE, and $\boldsymbol{X}_{A_k}$ and $\boldsymbol{X}_{B_k}$ are the corresponding initial coordinates; $\boldsymbol{I}$ is the identity tensor. Using two virtual nodes, associated to the top and bottom, and left and right edges, respectively, the following macroscopic deformation is prescribed:

$$\overline{F} = \begin{bmatrix} 1 - \sqrt{2E_{11} + 1} & 0 \\ 0 & UNSET \end{bmatrix},$$

corresponding to uniaxial tension or compression conditions (depending on the sign of $\overline{F}_{11}$), in which E_{11} is the uniaxial component of the Green-Lagrangian strain tensor, $\boldsymbol{E}$, used as a measure for finite strains. For more details on PBCs, please refer to [1].

Analytical model of wrinkling of interfacial layers

To demonstrate the ability of our model to predict complex mechanics, wrinkling of interfacial layers in stratified composites is considered. A hard interfacial layer is embedded in a compliant matrix subject to plain strain uniaxial compression. Here we report the dilute case i.e., “the matrix stress fields emanating from neighboring layers do not interact, hence the shear at the interface is negligible and interfacial layers behave independently of one another.” ref. 56 (main text); the long-wave instability is thus not here considered. Solving the governing equations for the critical instability conditions leads to the following expressions for the critical strain, ε_{cr}, and wavelength, λ_{cr} (ref. 56 main text):

$$\varepsilon_{cr} = C_\varepsilon(\nu_0)\left(\frac{E_1}{E_0}\right)^{-\frac{2}{3}},$$

$$\frac{\lambda_{cr}}{t} = C_\lambda(\nu_0)\left(\frac{E_1}{E_0}\right)^{\frac{1}{3}},$$

where C_ε and C_λ are coefficients that for plain strain read:

$$C_{\varepsilon}(\nu_0) = 2.08\left(\frac{3-4\nu_0}{(1-\nu_0)^2}\right)^{-\frac{2}{3}},$$
$$C_{\lambda}(\nu_0) = 2.18\left(\frac{3-4\nu_0}{(1-\nu_0)^2}\right)^{\frac{1}{3}}.$$

For strains higher than the critical one, the post-buckling wavelength, λ, and amplitude, A, can be obtained as (ref. 56 main text):

$$\lambda(\varepsilon) = \lambda_{cr}\, e^{-\varepsilon},\ \varepsilon > \varepsilon_{cr}$$

$$A(\varepsilon) = \frac{\lambda_{cr}}{\pi}\sqrt{|\varepsilon - \varepsilon_{cr}|},\ \varepsilon > \varepsilon_{cr}\ .$$

The plots in Fig. S7C-D are derived using these equations.

Details of the datasets

Lattice structures generation

To generate random finite-size lattice metamaterials, we adopt a bottom-up technique featured in our previous work (ref. 57 main text). With reference to Fig. S1, a squared building block of size s and beams' thickness t, and its $90°$-rotated version are combined into a 4×4 assembly i.e., the unit cell. A binary matrix is used to represent the unit cell, where 1 and 0 indicate either the first or the second building block. Random binary matrices thus correspond to random unit cells. In this work, we generate 762 different unit cells. Finally, to populate the dataset the resulting architectures are further tessellated to obtain 2×2 finite-size structures using $s = 2.5\ mm$ and a thicker frame surrounding the structures.

Details of the ML model set-up

GNN architecture

Our model consists of an encoder, a message-passing module, and a decoder (Fig. 1 main text). Let $\mathcal{G} = (\mathcal{V}, \mathcal{E})$ be a computational graph, where $\mathcal{V}$ represents a set of N nodes connected to each other through M edges ($\mathcal{E}$). The i-th node (in $\mathcal{V}$) brings n features in the vector $\boldsymbol{v}_i$ (such as nodal coordinates, and base material properties); similarly, the edge (in $\mathcal{E}$) connecting the i-th and j-th node has a m-dimensional feature vector $\boldsymbol{e}_{ij}$ (such as distance between nodes). The node and edge features, $\boldsymbol{v}_i$ and $\boldsymbol{e}_{ij}$, are encoded into a larger latent space in the encoder module using the two neural networks, ϵ^N and ϵ^E, respectively. The decoder is characterized by the neural network, δ^N, which transforms the latent node features to the output fields. Main core of the model is the message-passing module, which exploits the expressive power of GNNs. The message-passing phase runs for t from 1 to L steps. Defining the *node state*, $\boldsymbol{h}_i^t$, as the transformed latent node features after t message steps, messages are passed and aggregated at node i using the information from the neighboring nodes, $j \in \mathcal{N}(i) = \{j \in \mathcal{V} \mid (i,j) \in \mathcal{E}\}$, and corresponding edges, (i,j), as:

$$\boldsymbol{m}_i^{t+1} = \sum_{j \in N(i)} M^E(\boldsymbol{h}_i^t, \boldsymbol{h}_j^t, \boldsymbol{e}_{ij}^t)$$

where M^E is a neural network. The node state is then updated through the neural network U^N:

$$\boldsymbol{h}_i^{t+1} = U^N(\boldsymbol{h}_i^t, \boldsymbol{m}_i^{t+1})$$

Training of the model is supervised on nodal output fields by learning the parametrized differentiable functions ϵ^N, ϵ^E, M^E, U^N, and δ^N (i.e., neural networks) through minimization of a *mean absolute error* loss function.
For prediction of multiple loading steps (i.e., evolution of physical fields), to make the approach more general, we insert two gated recurrent units (GRUs)[2] after the message-passing module. The hidden states of the GRUs are collected and considered as the latent node state $\boldsymbol{h}_i^L$, then transformed by the decoder into output fields.

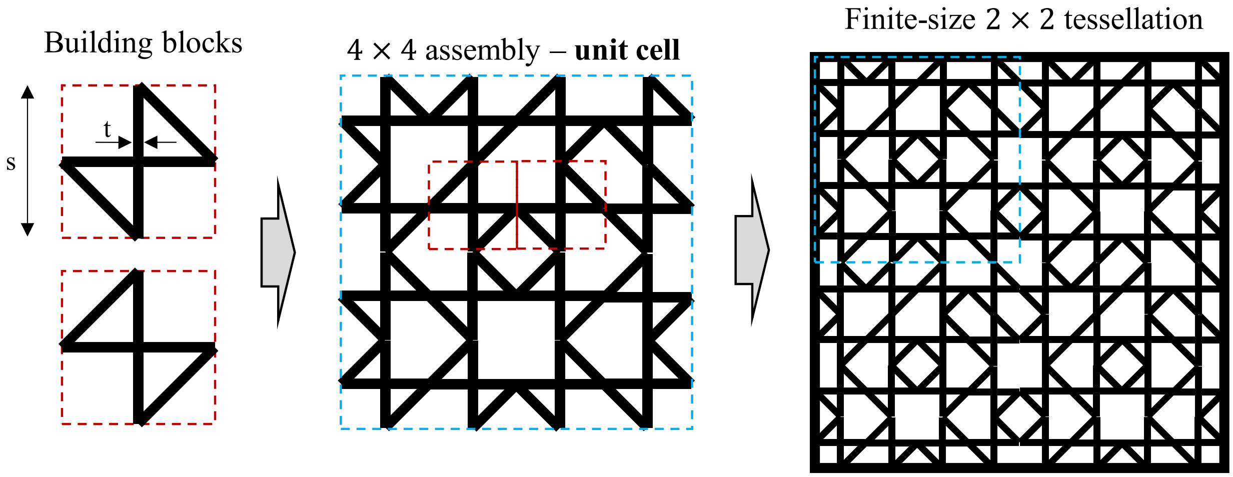

Fig. S1. Bottom-up technique of generation of finite-size lattice structures. Starting from two building blocks, a squared unit cell is first generated by randomly combining them. The dataset is populated by **2 × 2** tessellations of the randomly generated unit cells. The dashed boxes surrounding the building blocks and unit cell are color coded.

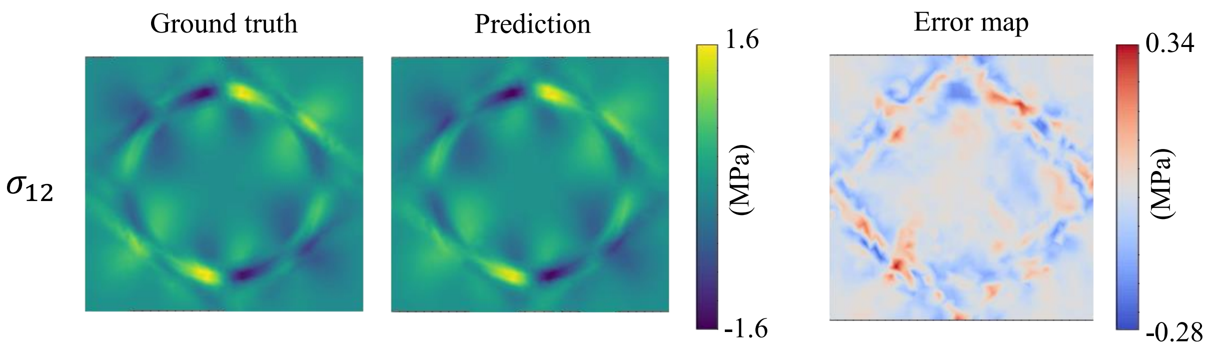

Fig. S2. Predicted vs. FE simulated shear stress field (σ_{12}) and error map in the fiber composite microstructure for $\bar{\varepsilon} = 6$ % shown in Fig. 2B-D.

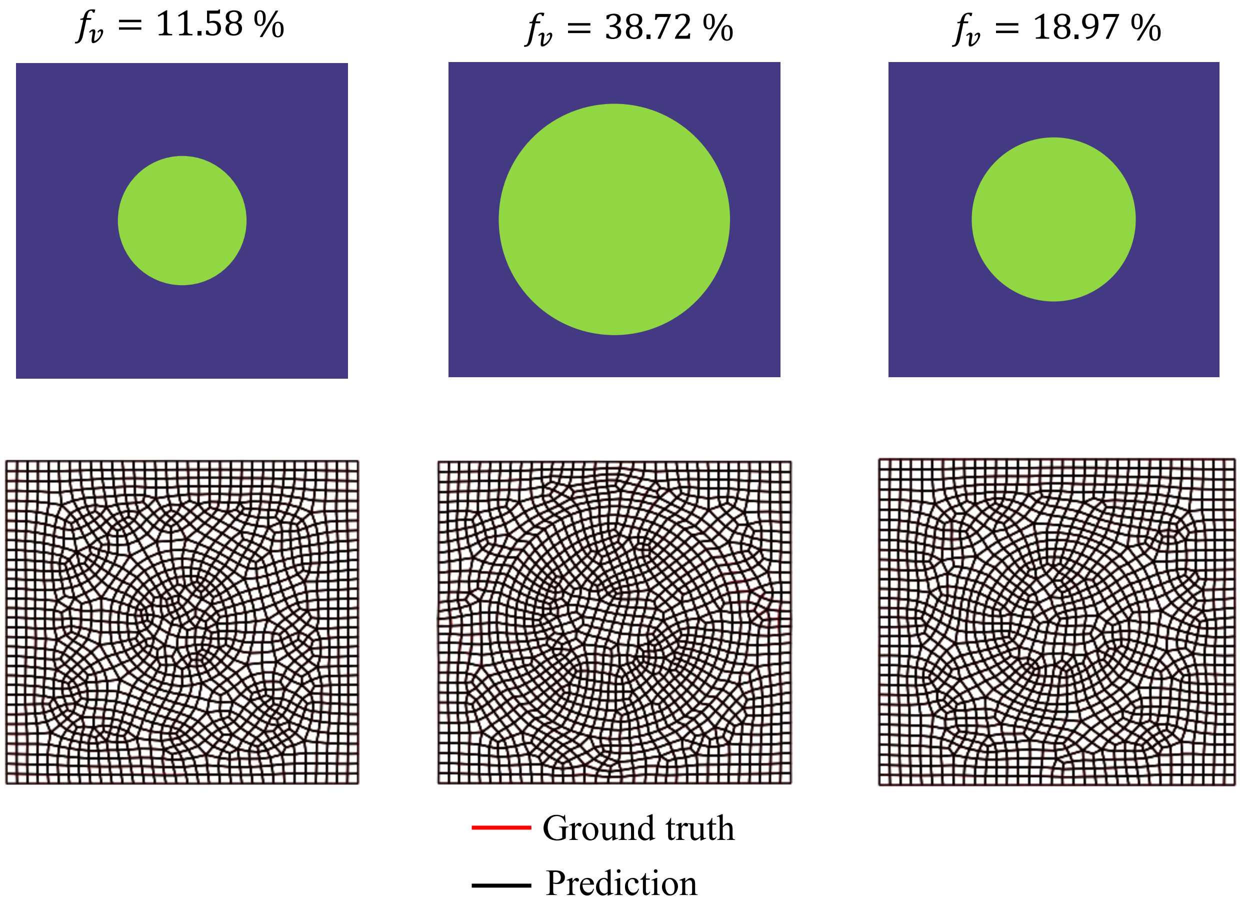

Fig. S3. Predicted vs. FE simulated deformations for $\bar{\varepsilon} = 6$ % of fiber composite microstructures with different volume fraction, randomly sampled from the test dataset.

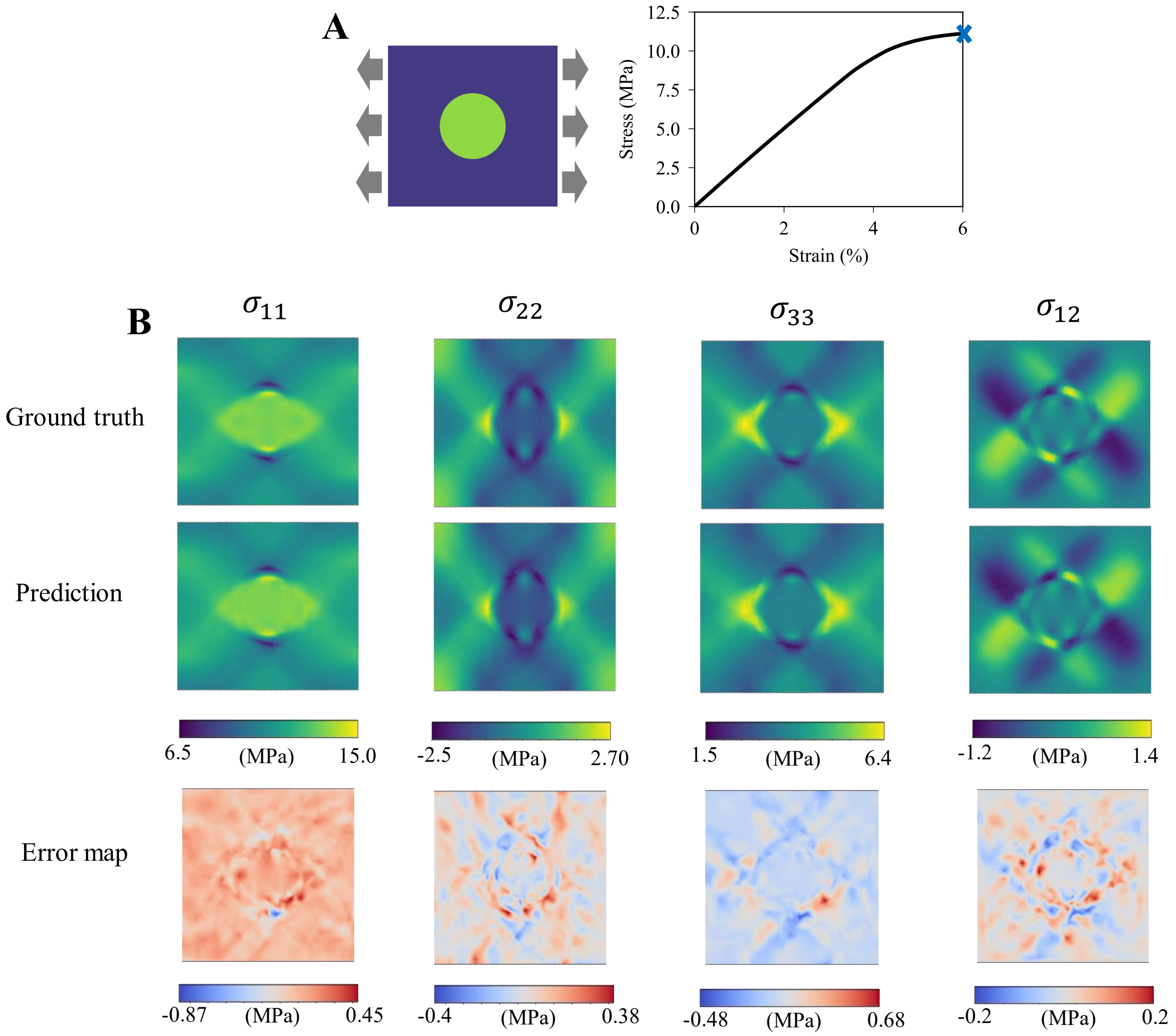

Fig. S4. Predicted vs. FE simulated deformation and stress fields for $\bar{\boldsymbol{\varepsilon}} = \mathbf{6}$ % in the first microstructure ($\boldsymbol{f_v} = \mathbf{11.58}$ %) of Fig. S3. (**A**) Representative boundary conditions together with the macroscopic stress-strain curve. The symbol on the plot identifies the large deformations regime ($\bar{\boldsymbol{\varepsilon}} = \mathbf{6}$ %). (**B**) Comparison of predictions and FE simulations for $\bar{\boldsymbol{\varepsilon}} = \mathbf{6}$ % in terms of deformation and stress fields together with the corresponding *error map*.

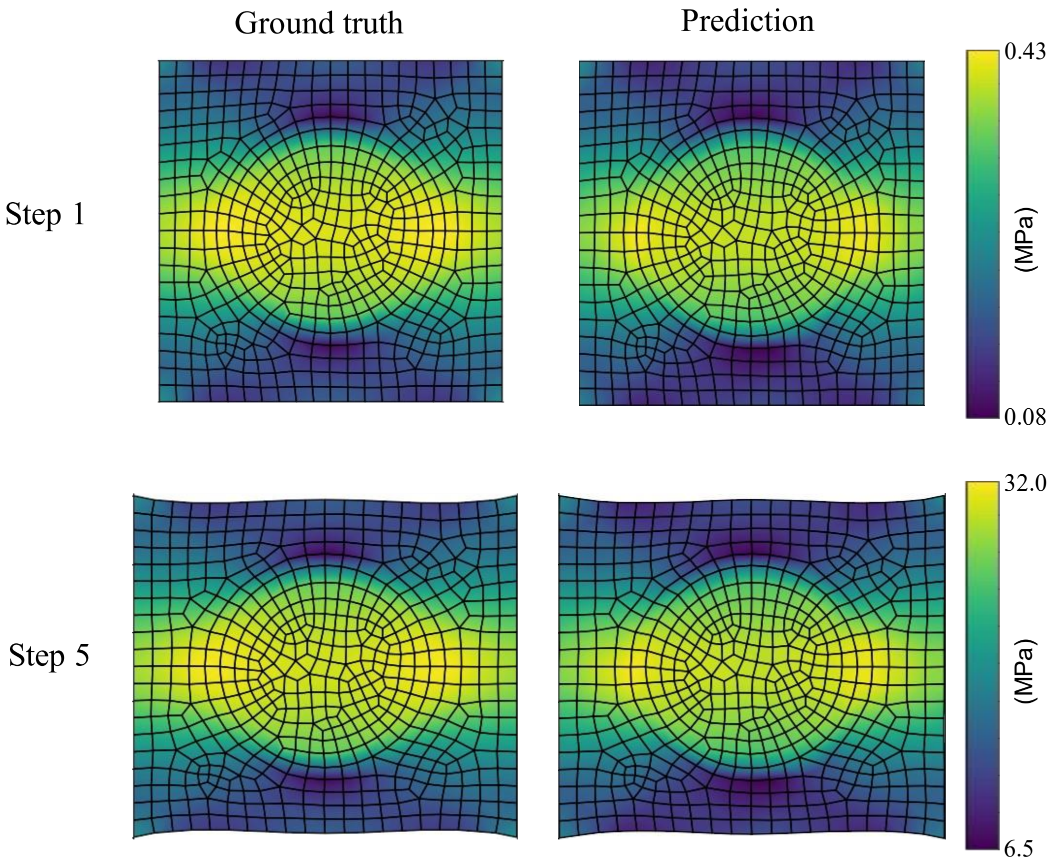

Fig. S5. Deformation and stress field evolution in fiber composite microstructures subject to displacement boundary conditions. Five loading steps, linearly sampled in the range 1 – 8 % of effective strain, are predicted. Here, comparison of FE simulations (i.e., ground truth) and ML predictions at the first and fifth loading step is shown.

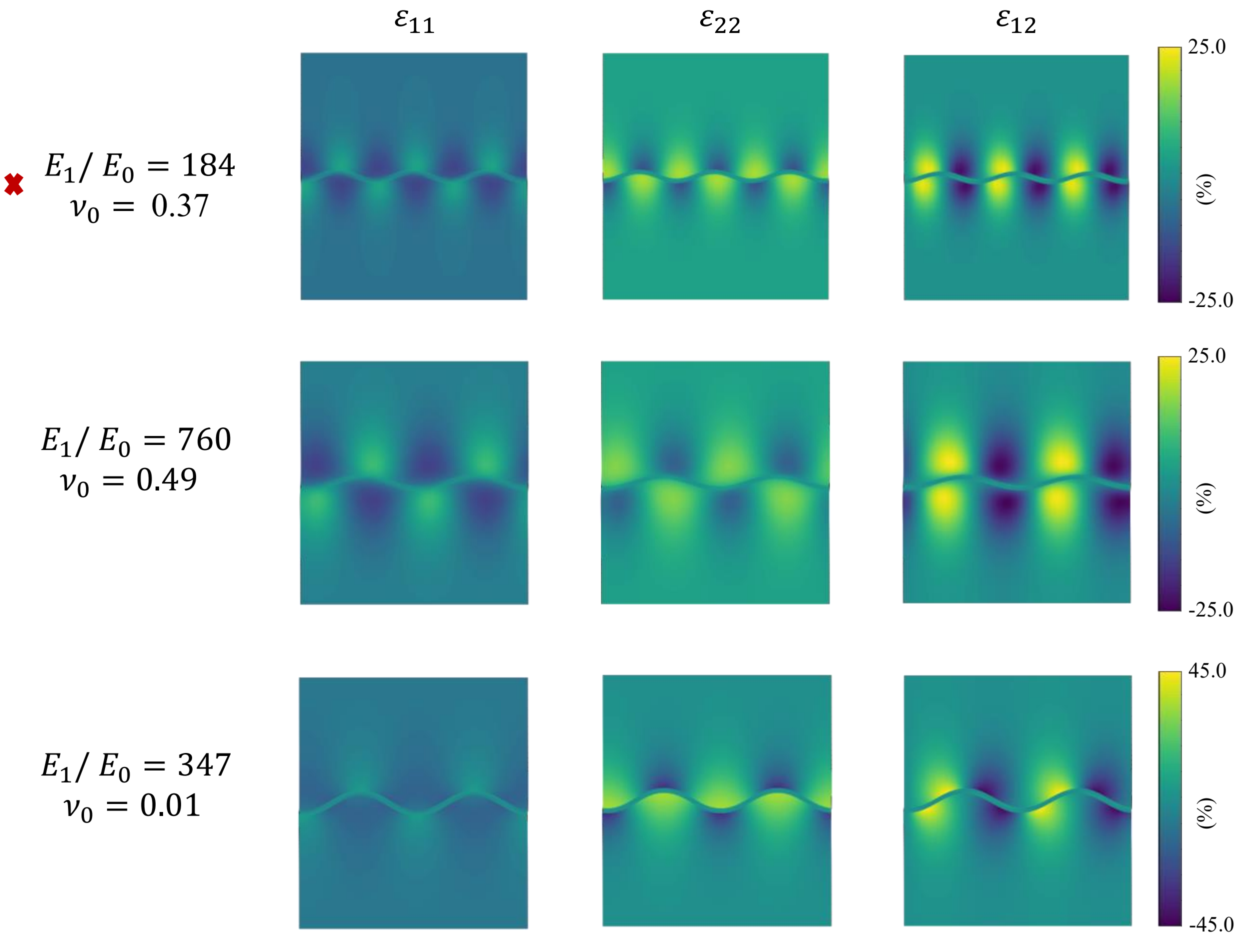

Fig. S6. FE simulated wrinkled interfacial layers in stratified composites for $\bar{\varepsilon} = 9$ % for different material property combinations randomly sampled from the test dataset. The cross in the first material property combination refers to the corresponding configuration in Fig. S7.

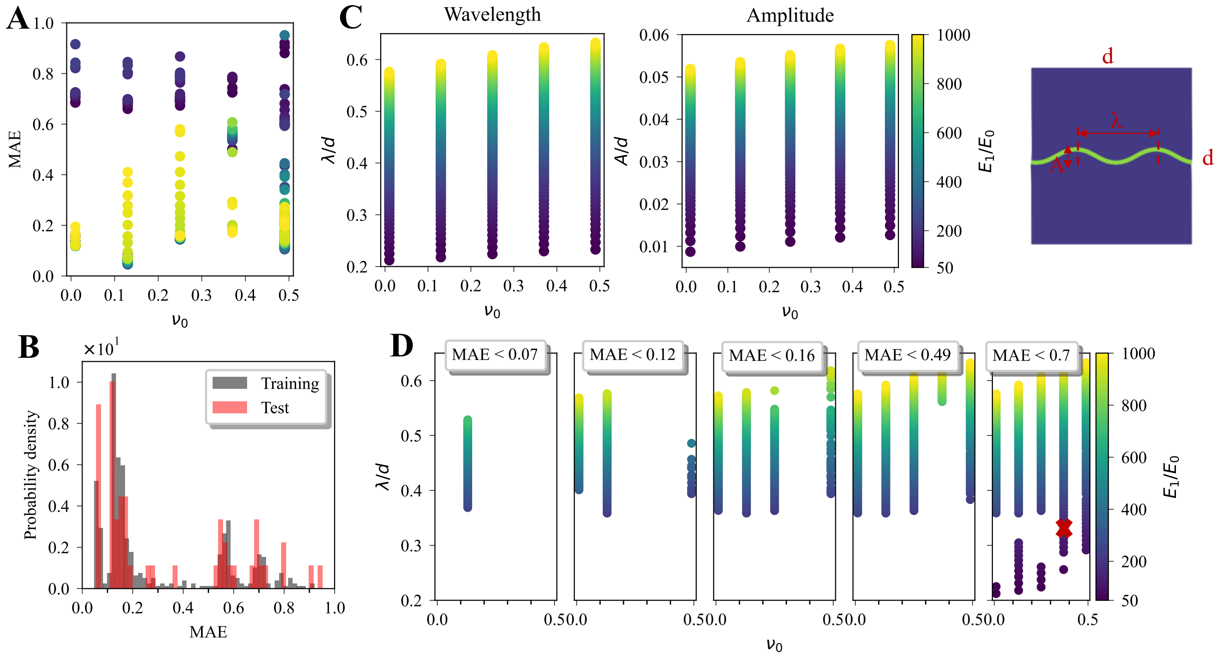

Fig. S7. Analysis of the predictive error of the GNN model on the wrinkling dataset. (A) Mean absolute error (MAE) vs. matrix Poisson's ratio (ν_0) for different stiffness ratios (E_1/E_0). **(B)** Probability density distribution of the MAE for the training and test dataset after pseudo-random shuffle with seed 42. **(C)** Analytical model results of wrinkling at $\bar{\varepsilon} = 9\ \%$. **(D)** Data in the first panel (wavelength) of **(C)** filtered by a threshold on the MAE. The cross in the last plot of **(D)** refers to the corresponding configuration in Fig. S6.

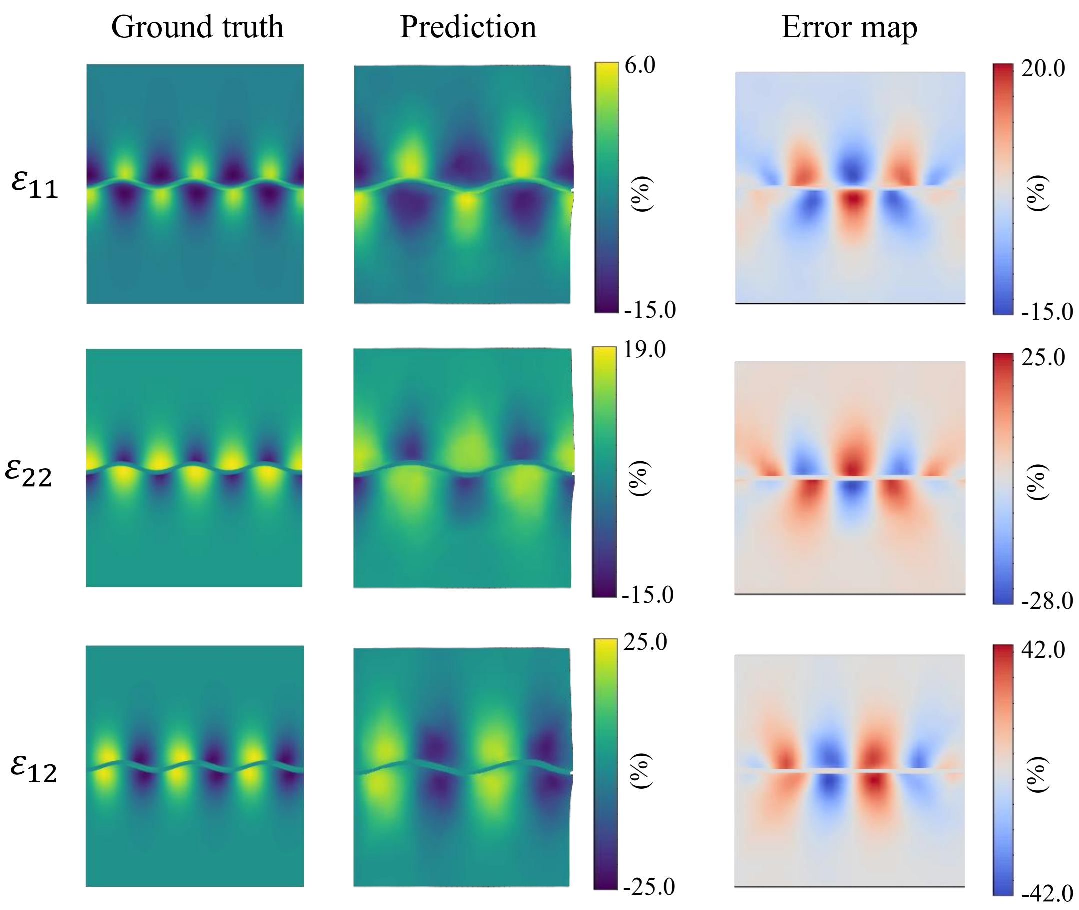

Fig. S8. Mismatch between predicted and FE simulated deformation and strain fields in the first configuration reported in Fig. S6-7 ($E_1/E_0 = 184$ and $\nu = 0.37$). Nonetheless, the model learns the relation between layer's curvature and strain pattern in cases of mismatched prediction.

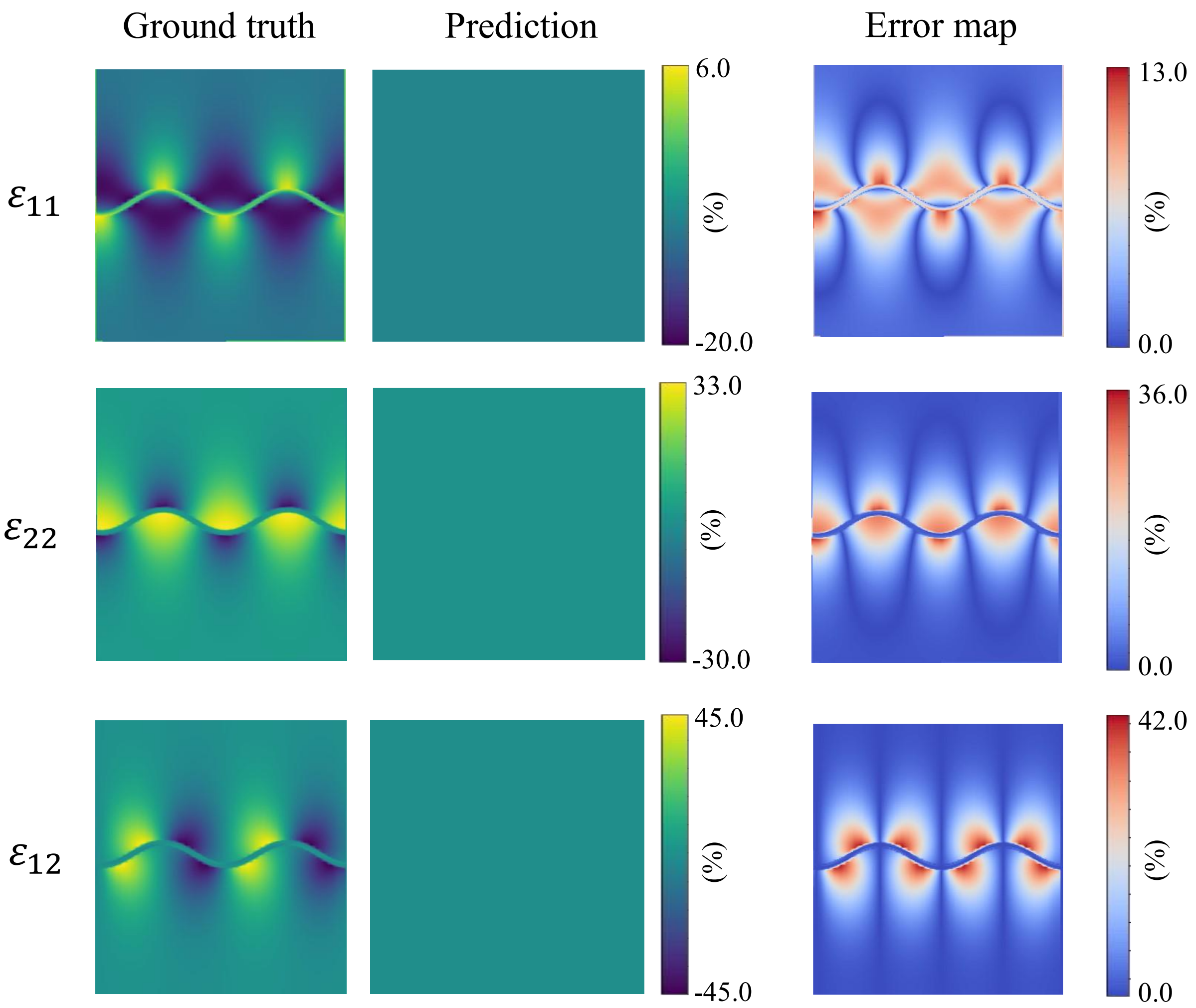

Fig. S9. U-Net predictions vs. FE simulations of the wrinkling problem at $\bar{\varepsilon} = 9$ % for the base material property configuration reported in Fig. 4 (main text). The U-Net architecture used here is similar to that adopted in ref. 48 main text (with classic convolutional layers); the code can be found in GitHub together with the GNN model (see main text).

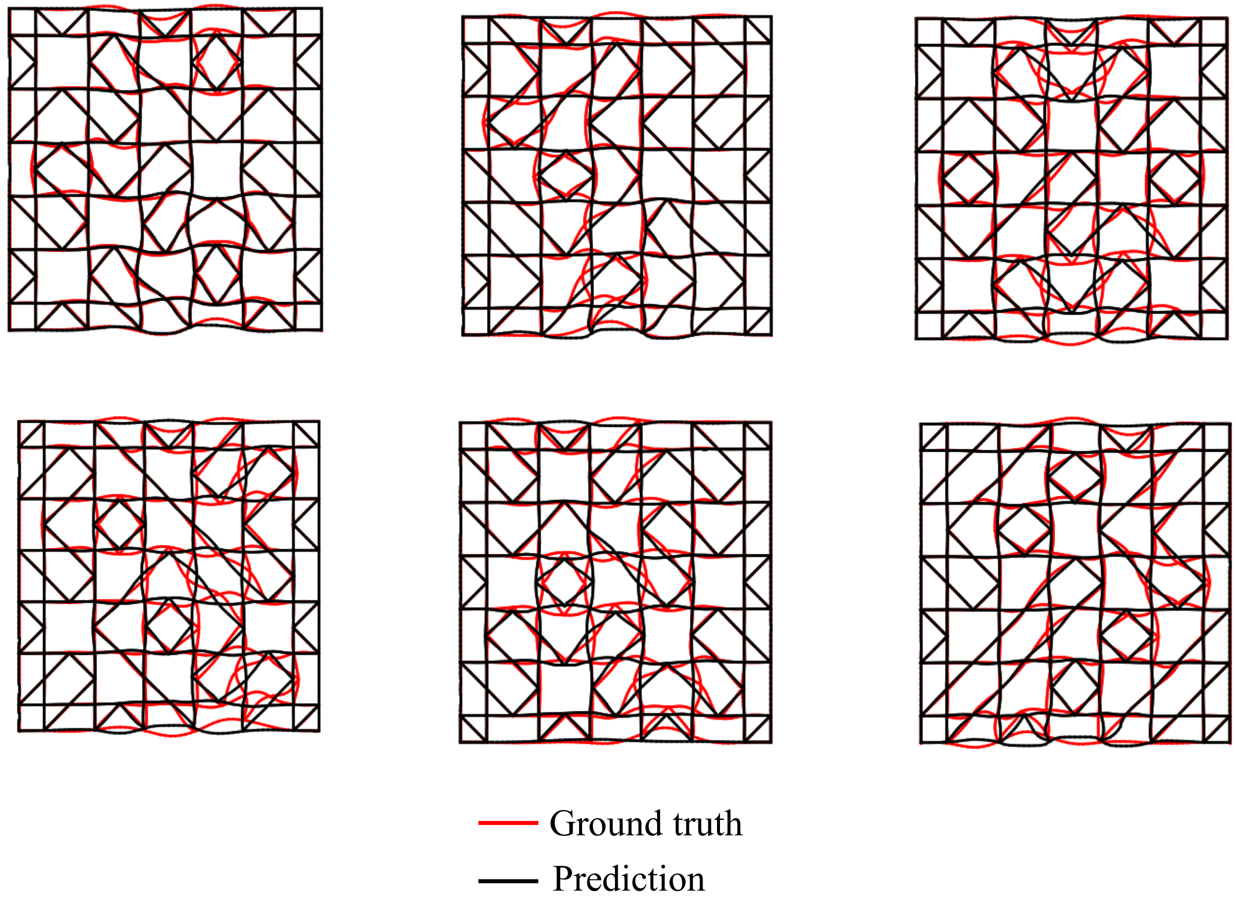

Fig. S10. Comparison of ML predicted and FE simulated post-buckled deformed shapes of hyperelastic lattice structures subject to uniaxial compressive loading with effective strain $\bar{\boldsymbol{\varepsilon}} = \mathbf{3}$ %. The six geometries are randomly extracted from the test dataset.

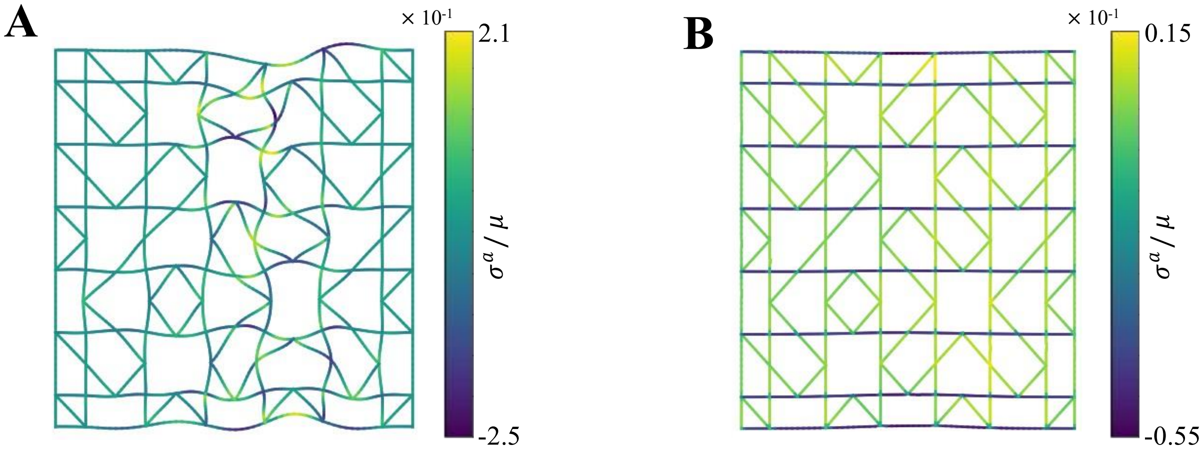

Fig. S11. Predicting buckling in lattice structures without providing the critical eigen-mode shape information to the ML model. (**A**) FE simulated post-buckled shape and stress field (axial stress on the lattice beams). (**B**) Corresponding ML prediction. The geometry is the first one in Fig. 4C (main text). Surprisingly, the model predicts a stress field resembling that exhibited before buckling instability occurs (see Fig. 4C main text). This leads to the observation that a physical relationship between global displacement (compression) and stress field is learned by the model.

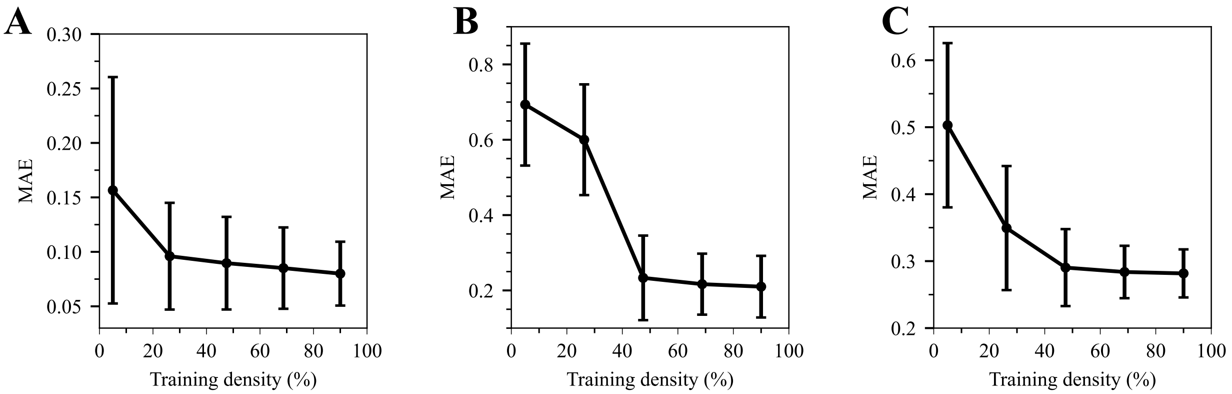

Fig. S12. Training data density sensitivity of the GNN model. (**A**) Plasticity in unidirectional fiber composites. (**B**) Wrinkling of interfacial layers in stratified composites. (**C**) Compression of architected lattice materials. Five training densities are linearly sampled in the range 5 – 90 %; for each value, 5 runs are carried out and the average MAE is evaluated on the test dataset. Random shuffling is repeatedly performed for each run. The error bars indicate the standard deviation.

Table S1. ML model hyperparameters for each dataset.

Datasets	Latent size	Message steps	Batch size	Training epochs
Fiber composites	128	10	1	100
Multi-step predictions	64	10	1	100
Stratified composites	32	15	2	100
Lattice structures	16	15	2	100

Table S2. Node and edge features, and output fields for each dataset; $\boldsymbol{x_{ij}} = \boldsymbol{x_i} - \boldsymbol{x_j}$.

Datasets	Node features	Edge features	Output fields
Fiber composites	$\boldsymbol{x}_i, \xi_i$	$\boldsymbol{x}_{ij}, \lvert\boldsymbol{x}_{ij}\rvert$	$\boldsymbol{u}_i, \boldsymbol{\sigma}_i$
Multi-step predictions	$\boldsymbol{x}_i, \xi_i, \boldsymbol{u}_i^{BC} or\ \boldsymbol{\varepsilon}_i^{BC}$	$\boldsymbol{x}_{ij}, \lvert\boldsymbol{x}_{ij}\rvert$	$\boldsymbol{u}_i, \boldsymbol{\sigma}_i$
Stratified composites	$\boldsymbol{x}_i, E_i, \nu_i$	$\boldsymbol{x}_{ij}, \lvert\boldsymbol{x}_{ij}\rvert$	$\boldsymbol{u}_i, \boldsymbol{\varepsilon}_i$
Lattice structures – small deformations	$\boldsymbol{x}_i$	$\boldsymbol{x}_{ij}, \lvert\boldsymbol{x}_{ij}\rvert$	$\boldsymbol{u}_i, \boldsymbol{\sigma}_i$
Lattice structures – large deformations	$\boldsymbol{x}_i, \widetilde{\boldsymbol{x}}_i$	$\boldsymbol{x}_{ij}, \widetilde{\boldsymbol{x}}_{ij}, \lvert\boldsymbol{x}_{ij}\rvert, \lvert\widetilde{\boldsymbol{x}}_{ij}\rvert$	$\boldsymbol{u}_i$

Table S3. Average computational time for data generation, training, and inference. Data generation time refers to the FE simulation and storage of the whole dataset; training time to a single epoch; inference time to a single microstructure. Training and inference time are evaluated using a GPU NVIDIA Quadro P2000. Training dataset size of 450 microstructures.

Datasets	Data generation (h)	Training (s)	Inference (s)
Fiber composites (single step)	8	75	0.015
Stratified composites	12.5	82	0.016
Lattice structures	12	9	0.009

Movie S1. Deformation and stress field evolution in a unidirectional fiber composite system under displacement boundary conditions.

Comparison between FE simulations (on the left) and ML predictions (on the right) of a single RVE subject to tensile displacement along the horizontal direction for five loading steps, from 1 to 8 % of effective applied strain. The ML model is trained and tested on the same five loading steps.

Movie S2. Deformation and stress field evolution in a unidirectional fiber composite system under displacement boundary conditions.

Comparison between FE simulations (on the left) and ML predictions (on the right) of a single RVE subject to tensile displacement along the horizontal direction for ten loading steps, from 1 to 8 % of effective applied strain. The ML model is trained on five loading steps and tested on ten steps (five from training).

1 Danielsson, M., Parks, D. & Boyce, M. Three-dimensional micromechanical modeling of voided polymeric materials. *Journal of the Mechanics and Physics of Solids* **50**, 351-379 (2002).

2 Cho, K., Van Merriënboer, B., Bahdanau, D. & Bengio, Y. On the properties of neural machine translation: Encoder-decoder approaches. *arXiv preprint arXiv:1409.1259* (2014).
